# Reactivity and increased proliferation of NG2 cells following central nervous system infection with Theiler’s murine encephalomyelitis virus

**DOI:** 10.1186/s12974-020-02043-5

**Published:** 2020-12-03

**Authors:** Laura A. Bell, Glenna J. Wallis, Karen S. Wilcox

**Affiliations:** 1 Department of Pharmacology and Toxicology, University of Utah, Salt Lake City, UT 84112 USA; 2 Interdepartmental Program in Neuroscience, University of Utah, Salt Lake City, UT 84112 USA

**Keywords:** Epilepsy, NG2, Glia, Gliosis, Microglia, Viral infection, Encephalitis, Theiler murine encephalomyelitis virus (TMEV)

## Abstract

**Background:**

Neuron-glial antigen 2 (NG2) cells are a glial cell type tiled throughout the gray and white matter of the central nervous system (CNS). NG2 cells are known for their ability to differentiate into oligodendrocytes and are commonly referred to as oligodendrocyte precursor cells. However, recent investigations have begun to identify additional functions of NG2 cells in CNS health and pathology. NG2 cells form physical and functional connections with neurons and other glial cell types throughout the CNS, allowing them to monitor and respond to the neural environment. Growing evidence indicates that NG2 cells become reactive under pathological conditions, though their specific roles are only beginning to be elucidated. While reactive microglia and astrocytes are well-established contributors to neuroinflammation and the development of epilepsy following CNS infection, the dynamics of NG2 cells remain unclear. Therefore, we investigated NG2 cell reactivity in a viral-induced mouse model of temporal lobe epilepsy.

**Methods:**

C57BL6/J mice were injected intracortically with Theiler’s murine encephalomyelitis virus (TMEV) or PBS. Mice were graded twice daily for seizures between 3 and 7 days post-injection (dpi). At 4 and 14 dpi, brains were fixed and stained for NG2, the microglia/macrophage marker IBA1, and the proliferation marker Ki-67. Confocal z stacks were acquired in both the hippocampus and the overlying cortex. Total field areas stained by each cell marker and total field area of colocalized pixels between NG2 and Ki67 were compared between groups.

**Results:**

Both NG2 cells and microglia/macrophages displayed increased immunoreactivity and reactive morphologies in the hippocampus of TMEV-injected mice. While increased immunoreactivity for IBA1 was also present in the cortex, there was no significant change in NG2 immunoreactivity in the cortex following TMEV infection. Colocalization analysis for NG2 and Ki-67 revealed a significant increase in overlap between NG2 and Ki-67 in the hippocampus of TMEV-injected mice at both time points, but no significant differences in cortex.

**Conclusions:**

NG2 cells acquire a reactive phenotype and proliferate in response to TMEV infection. These results suggest that NG2 cells alter their function in response to viral encephalopathy, making them potential targets to prevent the development of epilepsy following viral infection.

## Introduction

Temporal lobe epilepsy (TLE) is the most common form of acquired epilepsy and can develop following numerous insults to the central nervous system (CNS), including viral infection. Viral encephalitis is commonly associated with seizures and increases the risk of developing epilepsy by more than 20% over the general population [1–3]. A variety of neurotropic viruses cause encephalitis and are suggested to play a role in the development of seizures and epilepsy in humans, including human herpes virus (HHV), herpes simplex virus (HSV), Japanese encephalitis virus (JEV), Nipah virus, West Nile virus, enterovirus, Toscana virus, chikungunya virus, human immunodeficiency virus (HIV), influenza A and B, rotavirus, adenovirus, respiratory syncytial virus, cytomegalovirus, and severe acute respiratory syndrome coronavirus (SARS-CoV-2) [1–4].

The Theiler’s murine encephalomyelitis virus (TMEV) mouse model of TLE is the only well-characterized model of viral encephalitis-induced epilepsy that recapitulates the sequence of epilepsy pathogenesis seen in humans, including survival of the acute infection and a majority of animals that later develop spontaneous recurrent seizures after a seizure-free latent period. Intracortical injection of the Daniels’ strain of TMEV into a specific strain of mouse (C57BL/6 J) leads to acute encephalitic seizures from 3 to 8 days post-injection (dpi) [5, 6]. While several strains of TMEV have been shown to induce seizures in some strains of mice, the DA strain has been shown to induce the most severe and consistent seizures during the acute infection period [1, 7, 8]. Although the virus is cleared from the brain by around 14 dpi, a significant portion (> 50%) of mice goes on to later develop epilepsy starting around 2-months post-infection [5, 9, 10].

Approximately 30-40% of epilepsy patients remain refractory to therapy, indicating a substantial need to identify new targets for anti-seizure or anti-epileptogenic treatments [11–13]. While aberrant hyperexcitability and hypersynchronization of neurons are hallmarks of seizure activity, the presence of reactive glial cells or glial scar formation is nearly universal within epileptic lesions or foci. CNS glia, including astrocytes, microglia, neuron-glial antigen 2-expressing cells (NG2 cells), and oligodendrocytes are intricately involved in diverse neural functions, including neurotransmitter buffering, ion and water homeostasis, axon insulation, synapse function and plasticity, and blood-brain-barrier (BBB), extracellular matrix (ECM), and immune system regulation. It is now well-established that glial functions are commonly altered or disrupted in the process of epileptogenesis. Therefore, glial cells have come under recent focus as potential targets to promote homeostasis and functional recovery after a seizure or initiating epileptogenic event [14–18].

NG2 cells are a glial cell type abundantly tiled throughout the adult CNS and can be identified by the expression of the NG2 proteoglycan [also known as chondroitin sulfate proteoglycan 4 (CSPG4)]. Due to their capacity to generate and regenerate oligodendrocytes during development and adulthood, they are often referred to as oligodendrocyte precursor cells (OPCs). However, a growing body of evidence indicates much broader and more complex roles for NG2 cells in nervous system function and disease and has led to their classification as the fourth major glial cell type [19–22]. NG2 cells receive both inhibitory and excitatory synaptic inputs and express a distinguishing array of ion channels, receptors, and an immunoproteasome, uniquely priming them to monitor and respond to changes in the neural environment [21, 23–27].

In the TMEV model of TLE, TMEV infects hippocampal pyramidal neurons producing moderate to severe hippocampal necrosis and initiating seizure development [6, 9, 10, 28]. Glia cells, including astrocytes and microglia, respond to contain and to mitigate damage, but NG2 cell participation has not been determined. Previous studies from our lab have identified microglia as crucial regulators of the inflammatory response and astrocytes as active participants in synaptic remodeling following TMEV infection [5, 9, 28–30]. Reactive glia undergo metabolic and functional changes, including increased phagocytosis to clear debris, increased proliferation to amplify glial cell numbers, formation of a glial scar to seal off damage, increased production of proinflammatory cytokines to encourage defensive behaviors in neighboring cells, and morphologic hypertrophy by increasing glial cell size and/or surface protein expression. Therefore, in the present study, we investigated how NG2 cells respond to TMEV encephalitis at 4 and 14 days post-infection by examining NG2 cell hypertrophy, proliferation, and scar formation in the necrotic hippocampal region and in the non-directly affected cortex. Identifying the role of NG2 cells in epilepsy pathogenesis following viral encephalitis may lead to novel targets to block prolonged inflammatory response and to promote homeostasis and tissue repair.

## Materials and methods

### Animals

Male and Female C57BL/6 mice aged between 4 and 5 weeks old were purchased from Jackson Laboratory (Bar Harbor, ME, USA). Mice were acclimatized for 1 week prior to the experiments. Mice were provided food (Teklad Global Soy Protein-Free Extruded Rodent Diet cat. #2920X; Harlan Laboratories) and water ad libitum and housed in a temperature- and light-controlled (12 h light/dark cycle) environment. All procedures were performed in accordance with the National Institutes of Health Guide for the Care and Use of Laboratory Animals and approved by the Institutional Animal Care and Use Committee of the University of Utah.

### TMEV infection and seizure monitoring

Mice were anesthetized briefly using a mixture of isoflurane and compressed air. Mice were then injected with 20 μl of either phosphate-buffered saline (PBS, *n* = 8) or 2.5 × 10^5^ plaque forming units of Daniels strain of TMEV (*n* = 16) intracortically to a depth of 2 mm in the right hemisphere of the posterior parietal cortex as previously described [31–33]. Animals were monitored for handling-induced behavioral seizures and body weight changes twice daily from 3 to 7 dpi during their light cycle and between the hours of 9 am and 6 pm. Mice were agitated by briefly shaking their cages, and the intensity of the seizure activity was graded using a modified Racine scale as follows: stage 1, mouth and facial movements; stage 2, head nodding; stage 3, forelimb clonus; stage 4, forelimb clonus with rearing; stage 5, rearing and falling; stage 6, rearing and falling with extensive jumps or severe hind limb clonus [34]. Mice were included in histological analysis only if they were observed to have at least one seizure of a Racine score of 3 or greater. TMEV-infected mice and PBS controls were sacrificed at 4 dpi (PBS, *n* = 4; TMEV, *n* = 8) and 14 dpi (PBS, *n* = 4; TMEV, *n* = 8). These two time points were chosen as they represent times post-infection when animals begin to have seizures (4 dpi) and after seizures remit, and the virus is largely cleared (14 dpi). In addition, previous work from our lab has used those time points to assess microglia and astrocyte reactivity [33].

### Immunofluorescence

Animals were anesthetized through intraperitoneal injection with pentobarbital, and transcardial perfusions were performed with PBS followed by 10% neutral buffered formalin solution (NBF). Brains were then post-fixed 12-18 h in 10% NBF and transferred to a 15%/30% sucrose gradient for cryoprotection. Tissue was frozen and sectioned coronally to 30 μm on a freezing stage microtome (Leica, Buffalo Grove, IL). Slides were mounted with triplicate sections from both a PBS and TMEV brain as well as one section from each brain for a primary antibody-omission control to confirm antibody specificity on each slide. A hydrophobic barrier pen (Vector Laboratories H-4000) was used to separate the two control sections from the six stained sections. Slides were washed 3 times with PBS and blocked in CytoQ ImmunoDiluent & Block Solution (Innovex NB307-C) containing 0.3% Tween-20 for 1 h. Tissue was incubated overnight at 4 °C with primary antibodies directed to NG2 (Millipore/Sigma AB5230, 1:250), ionized calcium-binding adaptor molecule 1 (IBA1) (Novus Biologicals NB100-1028, 1:500), or Ki-67 (R&D Systems AF7649, 1:65) diluted in CytoQ ImmunoDiluent & Block Solution containing 0.05% Tween-20. Sections were then washed five times with CytoQ containing 0.1% Tween-20 and incubated for 2 h at room temperature with secondary antibodies, respectively, AlexaFluor donkey anti-rabbit 488, AlexaFluor donkey anti-goat 546, or AlexaFluor donkey anti-sheep 555. Slides were counterstained with DAPI (D9542, Sigma Aldrich). Slides were then rinsed five times with PBS and mounted with Prolong Gold antifade reagent (Molecular Probes) using No. 1.5 coverslips.

### Imaging and analysis

Images were captured with a Nikon A1 confocal microscope (Nikon Instruments, Melville, NY) at the University of Utah Cell Imaging Core Facility using a 20×/NA 1.0 air objective. Laser output, photomultiplier, and offset settings were adjusted to minimize saturated pixels and maximize contrast across samples. Once optimized, the laser settings were held constant between images acquired from control and TMEV-infected sections across all slides. Regions of interest, including the right dorsal CA1 region of the hippocampus and the overlying cortex, were initially identified using epifluorescence in the DAPI channel. Once a region was selected, laser-scanning mode was used to acquire 12 × 1 μm z stack optical images for each brain from triplicate sections using the Nikon’s Confocal NIS-Elements Acquisition Software.

Raw grayscale 12-bit images from the channel of each marker were processed to perform comparative fluorescence and colocalization analysis using the ImageJ software (National Institutes of Health, Bethesda, MD). Each image was first converted to 8-bit then processed using the “Stack Contrast Adjustment” plug-in by manually selecting the image with the highest contrast as the reference image for each stack [35]. An automated macro was created to perform the following functions to the stack contrast adjusted images in batch: A rolling ball background subtraction was performed and resulting images were processed using the “Hybrid 3D median Filter” plug-in [36]. Images containing NG2 and IBA1 stains were thresholded by setting a global threshold (value = 30). Images containing Ki-67 were thresholded at 0.35% stack histogram. For colocalization analysis, the corresponding images from each channel were processed through the “colocalization” plug-in [37]. Total field areas stained by each marker, as well as the total field area of the colocalized points between NG2 and Ki-67, were measured for each optical section through the stained tissue section. These values were averaged for each tissue section and then averaged across the triplicate sections stained from each brain.

For all representative images, brightness and contrast were adjusted linearly across the entire image to remove background and maximize contrast. For representative images showing comparison of IBA1 or NG2, the brightness and contrast levels for each channel were held constant between images. For representative images showing Ki-67, the Ki-67 channel was set to 0.35% histogram for each image to remove background and lower levels of expression in quiescent cells, such as neurons [38].

### Statistics

Total field area per stain was compared between brains from control and seized groups (PBS-injected vs. TMEV-injected) using two-tailed unpaired *t* tests. All data are presented with the mean value (*x*), the median value (middle line), and 25th to 75th percentiles. All statistical analysis was performed using MATLAB, and figures were prepared using Adobe Illustrator. *P* values less than 0.05 were considered statistically significant.

## Results

### Acute behavioral seizures in TMEV-infected mice

**Fig. 1:**
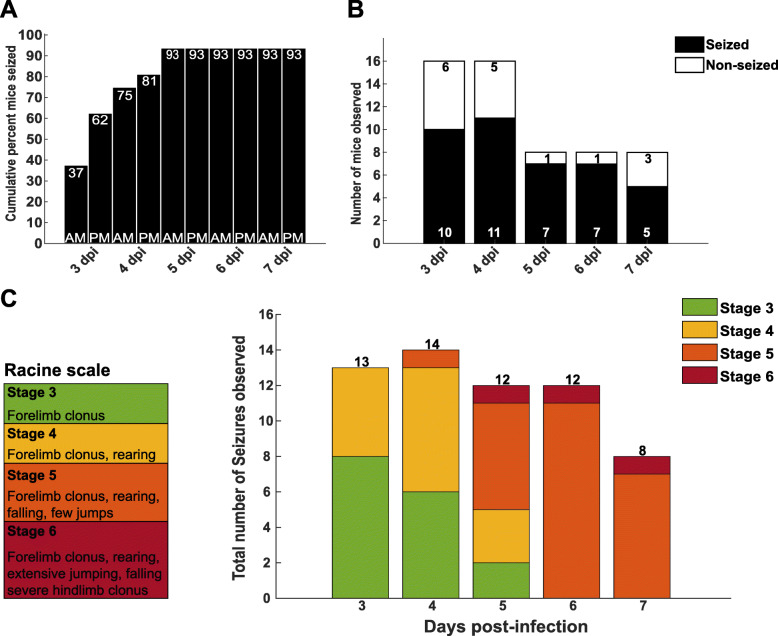
Intracranial injection of Theiler’s murine encephalomyelitis virus (TMEV) leads to acute behavioral seizures. **a** Cumulatively, 93.75% of the TMEV-infected animals exhibited at least one stage 3 or greater seizure during the twice daily (AM and PM) observation periods. Cumulative percentage of mice that experienced stage 3 or greater seizures listed on top of bars. **b** On any given day, the percentage of mice exhibiting a stage 3 or greater seizure ranged between 37 and 87% during any individual observation period. As half the total TMEV-infected mice (*n* = 16) were sacrificed after the observation period on 4 dpi, the number of mice observed from 5-7 dpi was reduced to *n* = 8. Number of seized and non-seized mice listed in bars. **c** The total numbers of seizures observed based on the modified Racine scale on each day during seizure monitoring show the seizure intensity increases over the course of the observation period. Number of seizures listed above bars

The majority of behavioral seizures occur between 3 and 7 dpi in the TMEV model [5]. Accordingly, we monitored behavioral seizures twice per day between 3 and 7 dpi. Because it is not possible to predict which animals will develop seizures, the number of animals was doubled for the TMEV group (*n* = 16). Cumulatively, 93.75% of TMEV-infected animals were observed to have at least one seizure prior to sacrifice (Fig. 1a). However, during the observation periods on any given day, the percentage of animals that showed stage 3 or greater seizure activity ranged from 37-87% (Fig. 1b). As previously described, seizure severity increased over the course of the observation periods, with the majority of seizures observed being either stage 5 or stage 6 by 6 dpi (Fig. 1c). Only animals that exhibited at least one stage 3 or greater seizure during the observation periods were used for further analysis. No seizures were observed in PBS-injected animals throughout the course of the study.

### NG2 cells become reactive in the hippocampus, but not the cortex, of TMEV-infected mice

**Fig. 2:**
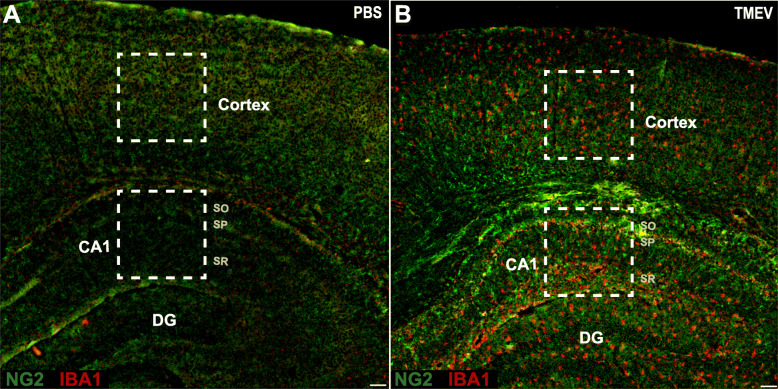
Imaging brain regions of interest. Low magnification (×4) confocal image showing NG2 (green) and microglia/macrophage marker ionized calcium-binding adaptor molecule 1 (IBA) (red) in a PBS-injected mouse brain (**a**) and a TMEV-infected mouse brain (**b**) at 4 dpi. White boxes identify representative imaging regions of interest in the cortex and the CA1 region of the hippocampus. SO, stratum oriens; SP, stratum pyramidal; SR, stratum radiatum. Scale bar = 100 μm

**Fig. 3:**
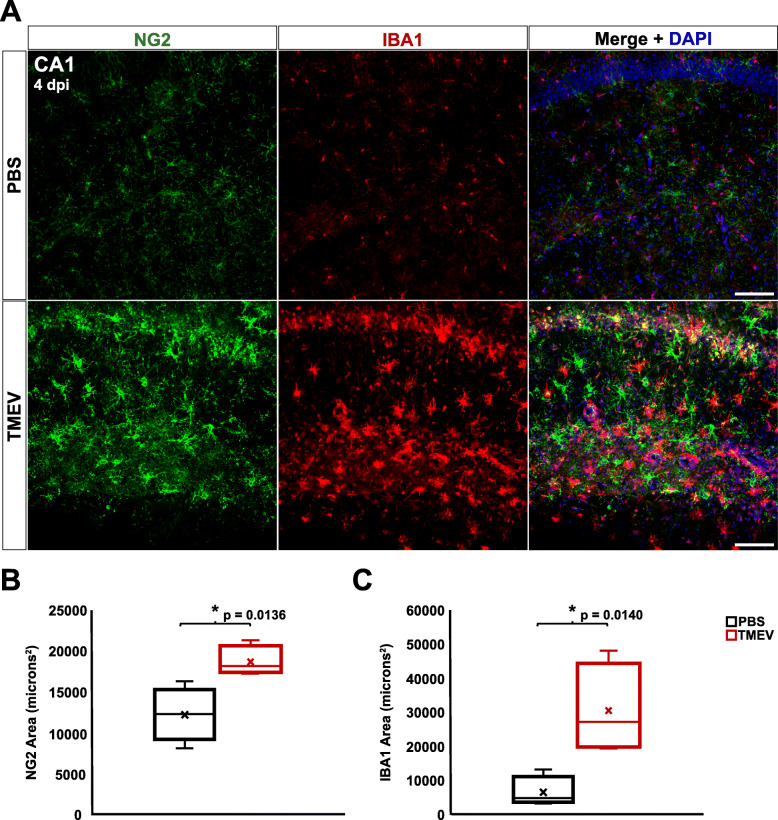
TMEV-injected animals exhibit reactive NG2 cells and microglia/macrophages in the hippocampus at 4 dpi. **a** NG2 cells (green) and microglia/macrophages (red) in the CA1 region of the hippocampus of PBS-injected (top) and TMEV-injected (bottom) mice at 4 dpi. **b** TMEV-injected mice showed significantly greater immunoreactivity for NG2 compared to PBS-injected mice. **c** TMEV-injected mice showed significantly greater immunoreactivity for IBA1 compared to PBS-injected mice. All images are ×20 maximum-intensity projections of 12 μm confocal z stacks. Scale bars = 100 μm

**Fig. 4:**
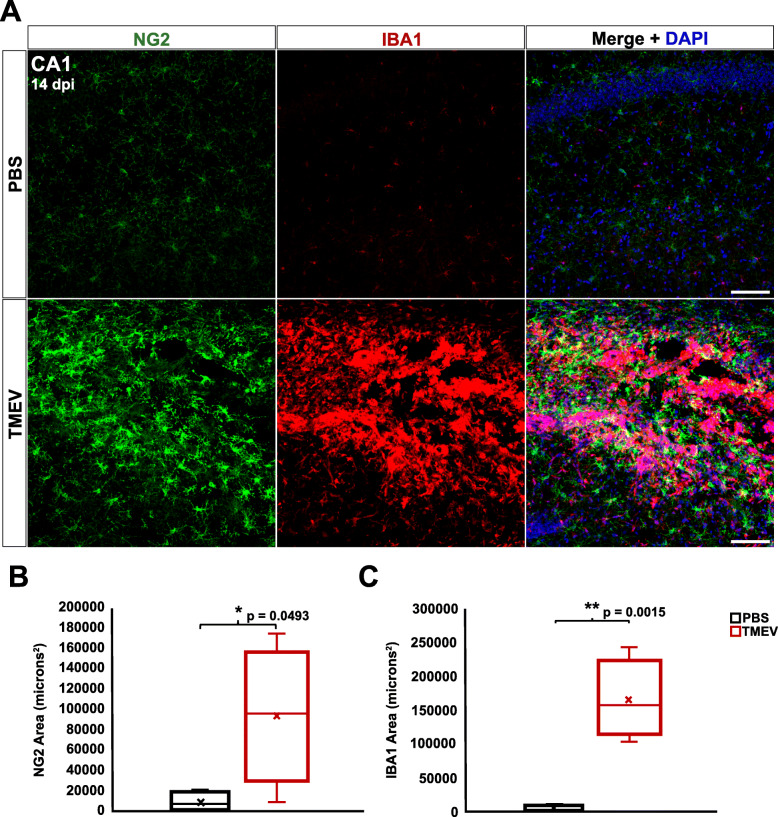
TMEV-injected animals exhibit scarring and reactive NG2 cells and microglia/macrophages in the hippocampus at 14 dpi. **a** NG2 cells (green) and microglia/macrophages (red) in the CA1 region of the hippocampus of PBS-injected (top) and TMEV-injected (bottom) mice at 14 dpi. **b** TMEV-injected mice showed significantly greater immunoreactivity for NG2 compared to PBS-injected mice. **c** TMEV-injected mice showed significantly greater immunoreactivity for IBA1 compared to PBS-injected mice. Images are ×20 maximum-intensity projections of 12 μm confocal z stacks. Scale bars = 100 μm

Following TMEV infection, reactive astrocytes and microglia/macrophages have been observed in the CA1 region of the hippocampus and in the cortex [33]. Reactivity of glial cells was also shown to persist well after viral clearance (≥ 14 dpi) [1, 5, 9, 31–33]. We aimed to build upon these findings and determine whether NG2 cells display a similarly reactive phenotype. We used immunohistochemistry at discrete time point’s post-TMEV infection to analyze immunoreactivity of anti-NG2 antibody for NG2 cells and anti-IBA1 antibody for microglia/macrophages. While IBA1 cannot discriminate between microglia and infiltrating macrophages, it does provide exceptional visualization of cell morphology. Therefore, quantitative analysis of IBA1 is used as a measure of changes in both the density and morphology of microglia as well as the infiltration of macrophages. Figure 2 shows wide-field examples of regions of interest in both a PBS-injected mouse (Fig. 2a) and a TMEV-infected mouse (Fig. 2b) at 4 dpi. We found the area positive for NG2 and IBA1 was significantly elevated in the CA1 hippocampal region of TMEV-injected mice at both 4 dpi (NG2 *p* = 0.0136 and IBA1 *p* = 0.0140) and 14 dpi (NG2 *p* = 0.0493 and IBA1 *p* = 0.0015) compared to PBS-injected controls (Figs. 3a-c and 4a-c). NG2 cell reactivity was also qualitatively confirmed based on morphological changes including hypertrophy of NG2 cell soma and processes (Figs. 3a and 4a) and their presence within the glial scar formation at 14 dpi (Fig. 4a).

**Fig. 5:**
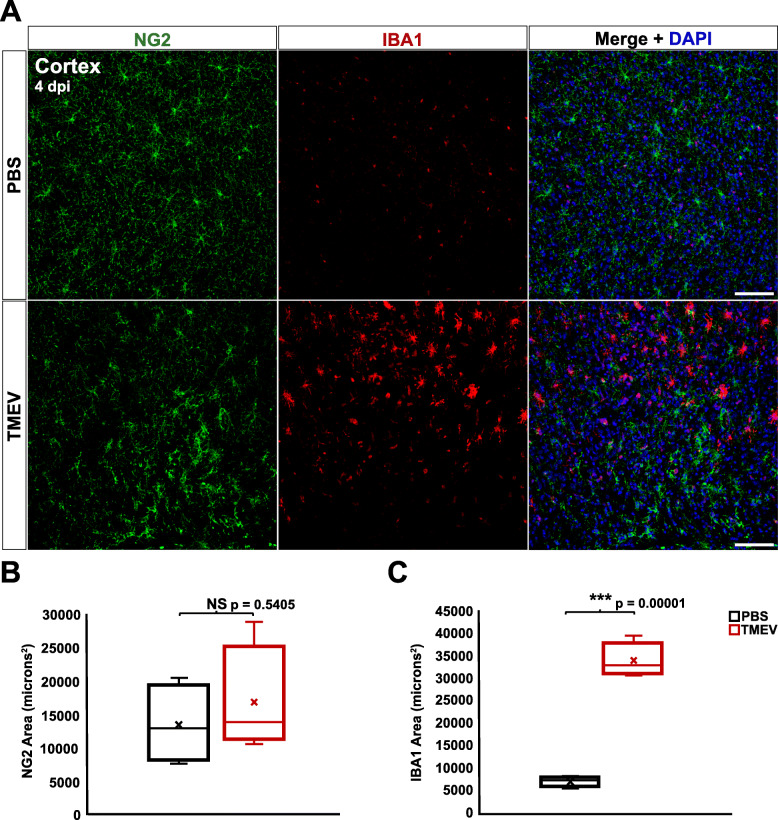
In contrast to microglia, NG2 cells in the cortex of TMEV-injected animals do not exhibit reactivity at 4 dpi. **a** NG2 cells (green) and microglia/macrophages (red) in the cortex of PBS-injected (top) and TMEV-injected (bottom) mice at 4 dpi. **b** TMEV-injected mice did not show significant changes in immunoreactivity for NG2 compared to PBS-injected mice. **c** TMEV-injected mice showed significantly greater immunoreactivity for IBA1 compared to PBS-injected mice. Images are ×20 maximum-intensity projections of 12 μm confocal z stacks. Scale bars = 100 μm

**Fig. 6:**
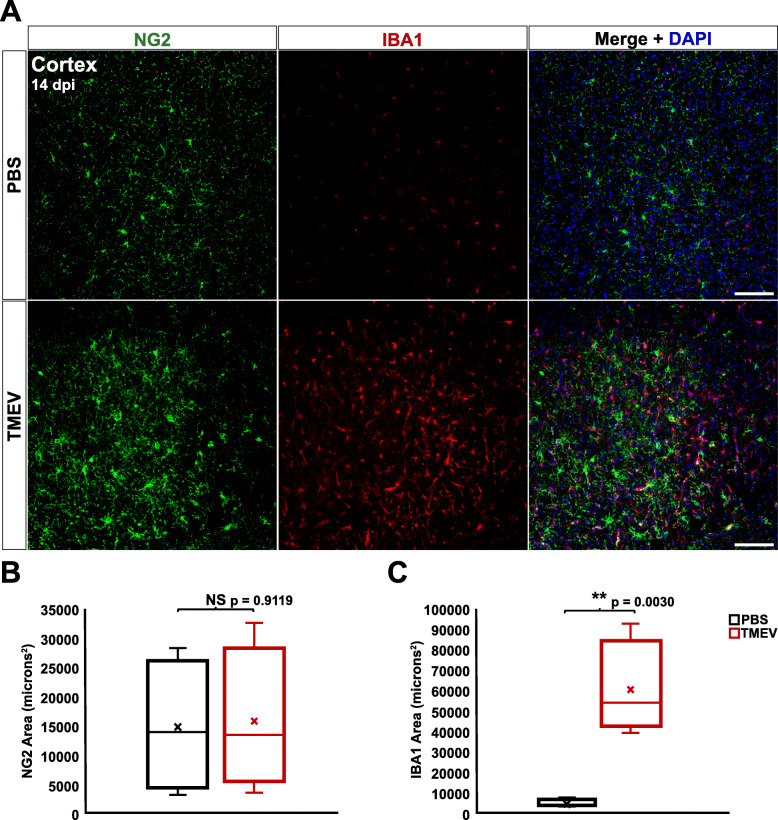
NG2 cells, in contrast to microglia, do not exhibit increased reactivity in the cortex at 14 dpi. **a** NG2 cells (green) and microglia/macrophages (red) in the cortex of PBS-injected (top) and TMEV-injected (bottom) mice at 4 dpi. **b** TMEV-injected mice did not show significant changes in immunoreactivity for NG2 compared to PBS-injected mice. **c** TMEV-injected mice showed significantly greater immunoreactivity for IBA1 compared to PBS-injected mice. Images are ×20 maximum-intensity projections of 12 μm confocal z stacks. Scale bars = 100 μm

In agreement with previous studies, significantly elevated IBA1 immunoreactivity was also present in the cortex of TMEV-infected mice compared to PBS-injected controls at both 4 dpi (*p* = 0.00001) (Fig. 5a, c) and 14 dpi (*p* = 0.0030) (Fig. 6a, c) [33]. However, analysis of NG2 immunoreactivity in the cortex revealed no significant difference between TMEV- and PBS-injected mice at either 4 dpi (*p* = 0.5405) (Fig. 5a-b) or 14 dpi (*p* = 0.9119) (Fig. 6a-b). These results suggest that NG2 cell reactivity is more localized to the hippocampal site of active infection and seizure focus compared to microglia/macrophages.

### Increased proliferation of NG2 cells in the hippocampus

**Fig. 7:**
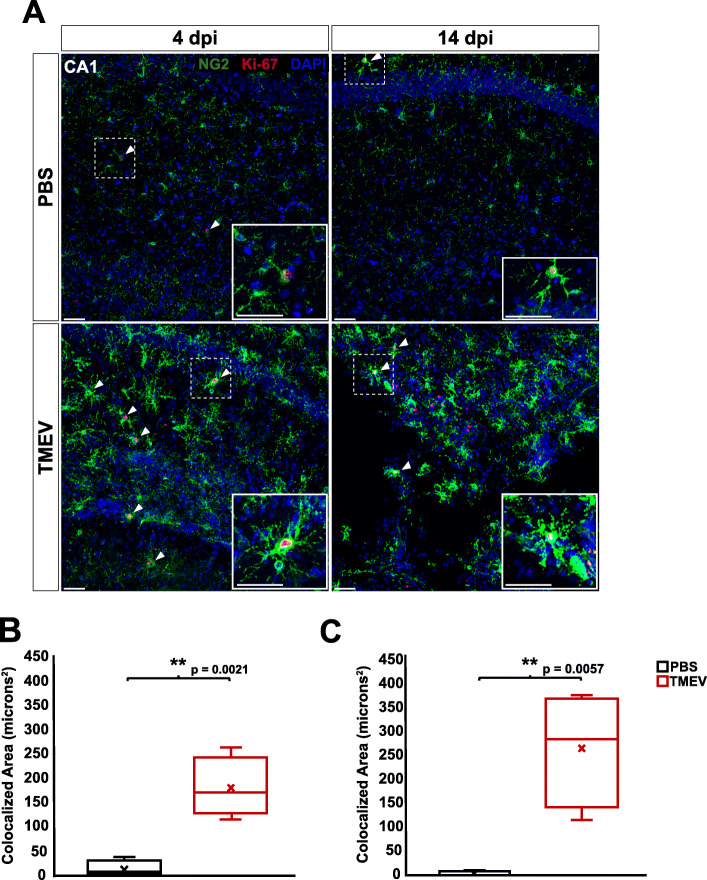
NG2 cell proliferation is increased in the hippocampus of TMEV-injected animals at 4 and 14 dpi. **a** NG2 cells (green) and proliferation marker Ki-67 (red) in the CA1 hippocampal region of PBS-injected (top) and TMEV-injected (bottom) mice at 4 and 14 dpi. Arrow heads point to cells containing pixels colocalized for both NG2 and Ki-67. **b** There is a significant increase in colocalization of NG2 and Ki-67 in the CA1 region of the hippocampus of TMEV-injected mice compared to PBS-injected mice at 4 dpi. **c** There is also a significant increase in colocalization of NG2 and Ki-67 in the CA1 region of the hippocampus of TMEV-injected mice compared to PBS-injected mice at 14 dpi. Images are ×20 maximum-intensity projections of 12 μm confocal z stacks. All scale bars = 50 μm

**Fig. 8:**
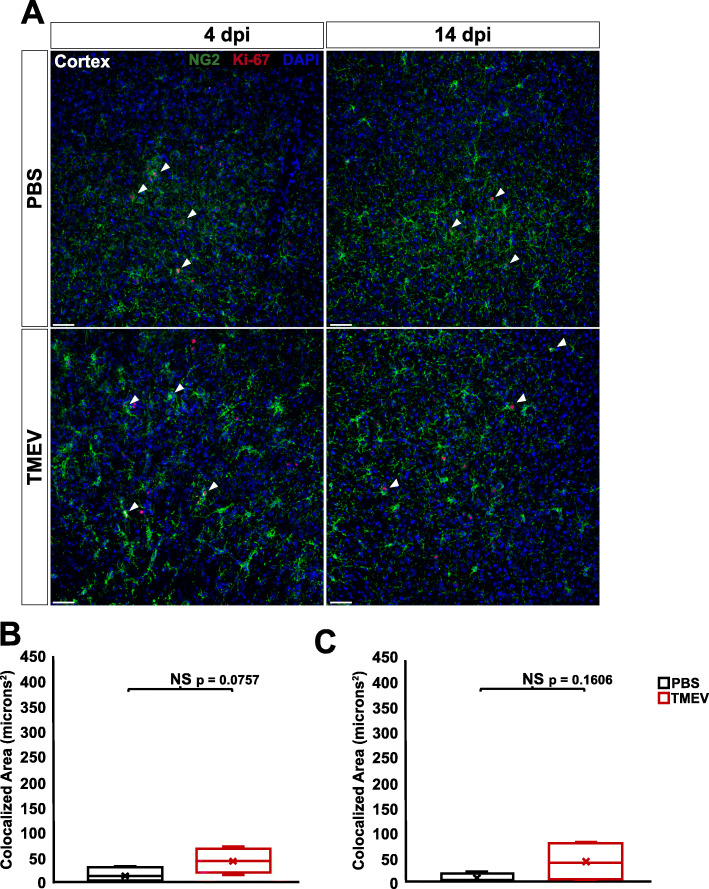
NG2 cell proliferation is not significantly changed in the cortex of TMEV-injected animals at both 4 and 14 dpi. **a** NG2 cells (green) and proliferation marker Ki-67 (red) in the cortex of PBS-injected (top) and TMEV-injected (bottom) mice at 4 and 14 dpi. Arrow heads point to cells containing pixels colocalized for both NG2 and Ki-67. **b** There is no significant change in colocalization of NG2 and proliferation marker Ki-67 in the cortex of TMEV-injected animals at 4 dpi. **c** There is also no significant change in colocalization of NG2 and Ki-67 in the cortex of TMEV-injected animals at 14 dpi. Images are ×20 maximum-intensity projections of 12 μm confocal z stacks. Scale bars = 50 μm

Glial activation and scar formation during CNS insult and epileptogenesis often coincide with increases in glial proliferation [17, 18, 33]. It was previously shown that both astrocytes and microglia undergo increased proliferation at 4 dpi in the TMEV model [33]. In order to determine whether NG2 cells also undergo increased proliferation in response to TMEV infection, we performed colocalization analysis to identify cells expressing both proliferation marker Ki-67 and NG2 [39]. A significant increase in overlap between NG2 and Ki-67 was observed at both 4 dpi (*p* = 0.0021) and 14 dpi (*p* = 0.0057) in the CA1 region of the hippocampus of TMEV-injected animals (Fig. 7a-c). The levels of colocalization between NG2 and Ki-67 did not differ significantly in the cortex between TMEV-injected animals and PBS-injected controls at either 4 dpi (*p* = 0.0757) or 14 dpi (*p* = 0.1606) (Fig. 8a-c). This data suggests that there is ongoing increased proliferation of NG2 cells in the hippocampus, but not cortex, following TMEV infection.

## Discussion

This study is the first to evaluate the effect of TMEV infection and seizures on the activation state of NG2 cells using IHC techniques. Quantitative analysis revealed reactivity of both NG2 cells and microglia/macrophages in the CA1 region of TMEV-injected brains at 4 and 14 dpi as measured by significantly greater immunoreactivity of NG2 and IBA1, respectively. Our data adds NG2 cells to the repertoire of reactive cell types and participants in glial scar formation in response to TMEV infection and replicates previous studies identifying reactive microglia/macrophages in both the hippocampus and cortex [33]. NG2 cells express a wide array of Ca^2+^ channels and receptors that regulate diverse aspects of NG2 cell morphology and function, including immune signaling, process dynamics, cell migration, intercellular communication, and release of trophic and secreted factors [27]. Reactive NG2 cells displayed characteristic thick and ramified processes, reinforcing the possibility that NG2 cells, like other glia, likely undergo altered gene expression and physiological changes that coincide with the observed morphological changes following CNS infection [26, 27, 40, 41].

We also observed increased proliferation of NG2 cells in the CA1 region of TMEV-infected brains. While NG2 cells are the most proliferative cells in the healthy adult brain, increased NG2 cell proliferation has been demonstrated following several types of CNS injury [42–46]. It is noteworthy that, unlike microglia, enhanced NG2 cell reactivity and proliferation appeared to localize to the CA1 region of the hippocampus, the site of active infection, and significant neuronal cell death in the TMEV-model [5, 6, 33]. Additionally, while previous studies found that initial increases in proliferation of microglia/macrophages and astrocytes diminished to undetectable levels by 14 dpi, increased proliferation of NG2 cells observed at 4 dpi was sustained at the 14 dpi time point [33]. This prolonged increase in proliferation suggests that NG2 cells may play a role in the ongoing innate immune response and scar formation following TMEV infection.

Glial cells dynamically sense and respond to the neural environment. Upon an initiating neurological insult or injury, reactive astrocytes, microglia, and NG2 cells have been demonstrated to secrete pro-inflammatory cytokines in order to adapt to damage and promote homeostasis. However, in cases of chronic epilepsy, the release of pro-inflammatory cytokines can become harmful, contributing to neuronal hyperexcitability and the generation of seizures [47, 48]. Several studies have identified immune antigen-presentation on NG2 cells in response to neurological insult or injury, suggesting NG2 cells may play a role in the inflammatory milieu contributing to seizures following TMEV infection [7, 23, 29, 34, 49]. Conversely, NG2 cells have also been shown to suppress neuroinflammation and microglial activation through TGF-β signaling [26]. These diametric findings suggest that reactive NG2 glia may assume immune-promoting or immune-suppressing roles in a context-specific manner. Untangling the signaling pathways involved in NG2 cell immunomodulation may provide new therapeutic targets aimed at tipping the balance of an uncontrolled or prolonged neuroimmune response following viral infection.

Several reports also provide evidence that reactive NG2 cells within the vicinity of injury sites function to form a barrier around damaged tissue in order to protect the surrounding healthy tissue [44, 50]. A recent study by Damisah et al. demonstrates that microglia, astrocytes, and NG2 cells coordinate their response to clear damaged and dying neurons, with NG2 cells not only polarizing toward dying neurons but remaining to fill the lesion after removal of neuronal debris was complete [51]. While the exact purpose of NG2 cell activity in the formation of the glial scar remains unclear, increased expression, or deposits of NG2, otherwise known as chondroitin sulfate proteoglycan 4 (CSPG4), may form a barrier to repair damage in epilepsy-inducing lesions and significantly alter the function of surviving neurons and astrocytes, contributing to epileptogenesis [18]. Because NG2 cell reactivity is spatially restricted to the site of active infection and seizure focus, deciphering their specific functions within the scar could provide novel insights into mechanisms underlying epileptogenesis.

NG2 cell reactivity, proliferation, and involvement in scar formation are present within inflammatory lesions in the spinal cord of TMEV-infected SJL/J mice. In contrast to the TMEV model of TLE, intracerebral infection of a seizure-resistant SJL/J mouse strain causes acute encephalitis followed by viral persistence and chronic inflammatory demyelinating disease predominantly in the spinal cord. Accordingly, TMEV-infected SJL/J mice are a widely used animal model of multiple sclerosis [1, 8, 52]. During chronic demyelination, NG2 cells are shown to exhibit alternate differentiation pathways where proliferating NG2 cells shift their differentiation pattern from an oligodendrocytic to an astrocytic fate, which has been implicated as a major factor in disease progression and incomplete remyelination [53]. While C57BL/6 mice in the TMEV model of TLE do not exhibit spinal cord pathology, understanding the cell signaling pathways involved in NG2 cell proliferation, density, and differentiation that contribute to inflammation and scar formation may similarly be important in understanding the impedance for tissue regeneration and repair in the inflamed hippocampus of TMEV-infected C57BL/6 mice [10].

Together, our data show robust NG2 cell activation and proliferation in response to TMEV-infection and acute seizures. NG2 cells are increasingly being recognized as important players in promoting CNS health and pathology. While the exact function of NG2 cell reactivity in response to TMEV infection is still unknown, the combination of advanced RNA-sequencing technologies along with functional imaging approaches using genetically encoded calcium indicators can be used to target the role of NG2 cells in the process of epileptogenesis. While most currently available anti-seizure drugs target neuronal mechanisms, 30-40% of epilepsy patients remain refractory to currently available therapies. Identifying the role of NG2 cells in epilepsy pathogenesis following viral encephalitis may lead to novel targets and strategies toward blocking prolonged inflammatory response and facilitating therapies designed to promote homeostasis and tissue repair.

## Conclusions

In summary, our study identifies reactive NG2 cells in the inflamed and epileptogenic hippocampus following CNS infection with TMEV, indicating that NG2 cells are involved in the neuroinflammatory response and may be promising targets for preventing seizures and epilepsy following viral infection.

## Data Availability

The datasets used and/or analyzed in the present study are available from the corresponding author upon reasonable request.

## Abbreviations

NG2: Neuron-glial antigen 2
CNS: Central nervous system
TMEV: Theiler’s murine encephalomyelitis virus
PBS: Phosphate-buffered saline
NBF: Neutral buffered formalin
Dpi: Days post-injection
IBA1: Ionized calcium-binding adaptor molecule 1
TLE: Temporal lobe epilepsy
HHV: Human herpes virus
HSV: Herpes simplex virus
JEV: Japanese encephalitis virus
HIV: Human immunodeficiency virus
SARS-CoV-2: Severe acute respiratory syndrome coronavirus
BBB: Blood-brain barrier
ECM: Extracellular matrix
OPC: Oligodendrocyte precursor cell
IHC: Immunohistochemistry
CA1: Cornu Ammonnis subfield 1
TGF-β: Transforming growth factor beta
CSPG4: Chondroitin sulfate proteoglycan 4
